# Building better systems of care for Aboriginal and Torres Strait Islander people: findings from the Kanyini health systems assessment

**DOI:** 10.1186/1472-6963-12-369

**Published:** 2012-10-28

**Authors:** David Peiris, Alex Brown, Michael Howard, Bernadette A Rickards, Andrew Tonkin, Ian Ring, Noel Hayman, Alan Cass

**Affiliations:** 1 The George Institute for Global Health, University of Sydney, Sydney, Australia; 2 The Baker IDI Heart and Diabetes Institute, Alice Springs, Australia; 3 Menzies School of Health Research, Adelaide, Australia; 4 Monash University, Melbourne, Australia; 5 University of Wollongong, Wollongong, Australia; 6 Inala Indigenous Health Service, Brisbane, Australia

## Abstract

**Background:**

Australian federal and jurisdictional governments are implementing ambitious policy initiatives intended to improve health care access and outcomes for Aboriginal and Torres Strait Islander people. In this qualitative study we explored Aboriginal Medical Service (AMS) staff views on factors needed to improve chronic care systems and assessed their relevance to the new policy environment.

**Methods:**

Two theories informed the study: (1) ‘candidacy’, which explores “the ways in which people’s eligibility for care is jointly negotiated between individuals and health services”; and (2) *kanyini* or ‘holding’, a Central Australian philosophy which describes the principle and obligations of nurturing and protecting others. A structured health systems assessment, locally adapted from Chronic Care Model domains, was administered via group interviews with 37 health staff in six AMSs and one government Indigenous-led health service. Data were thematically analysed.

**Results:**

Staff emphasised AMS health care was different to private general practices. Consistent with *kanyini*, community governance and leadership, community representation among staff, and commitment to community development were important organisational features to retain and nurture both staff and patients. This was undermined, however, by constant fear of government funding for AMSs being withheld. Staff resourcing, information systems and high-level leadership were perceived to be key drivers of health care quality. On-site specialist services, managed by AMS staff, were considered an enabling strategy to increase specialist access. Candidacy theory suggests the above factors influence whether a service is ‘tractable’ and ‘navigable’ to its users. Staff also described entrenched patient discrimination in hospitals and the need to expend considerable effort to reinstate care. This suggests that Aboriginal and Torres Strait Islander people are still constructed as ‘non-ideal users’ and are denied from being ‘held’ by hospital staff.

**Conclusions:**

Some new policy initiatives (workforce capacity strengthening, improving chronic care delivery systems and increasing specialist access) have potential to address barriers highlighted in this study. Few of these initiatives, however, capitalise on the unique mechanisms by which AMSs ‘hold’ their users and enhance their candidacy to health care. *Kanyini* and candidacy are promising and complementary theories for conceptualising health care access and provide a potential framework for improving systems of care.

## Background

The Australian federal government has recently launched several policy initiatives to improve access to quality health care for the prevention and management of chronic diseases for Aboriginal and Torres Strait Islander people. In November 2008 the Council of Australian Governments established a National Partnership Agreement on ‘Closing the Gap in Indigenous health outcomes’ (henceforth termed the “COAG NPA”). In addition to other agreements on housing and employment, the COAG NPA, budgeted at $1.6 billion over four years, has created unprecedented levels of funding for health service initiatives. The $800 million federal government component is focussed on improving outcomes for chronic diseases [1]. The package has three components: (1) ‘*tackling chronic disease risk factors’* focuses on reducing smoking prevalence, the creation of an Aboriginal and Torres Strait Islander tobacco and lifestyle workforce, and use of social marketing campaigns to promote better health; (2) *‘improving chronic disease management and follow-up care’* focuses on improved access to medicines, incentives to care providers to improve chronic care management, improved care coordination and access to specialist services, and monitoring and evaluation of all these initiatives; and (3) ‘*workforce expansion’* focuses on increasing primary care workforce capacity, the use of Aboriginal and Torres Strait Islander outreach workers to broker health services, additional nursing and general practitioner (GP) training placements and promotion of guideline-based care. In our research we sought to better understand Aboriginal Medical Services’ (AMSs) perspectives on health systems barriers and enablers and assess their relevance to these policy initiatives. Although AMSs can be either community governed (known as Aboriginal Community Controlled Health Services) or run by State or Territory governments, the term is most commonly applied to the former [2]. We build on the work of two research projects. The Improving Access to Kidney Transplants qualitative study examined patients, staff and policy makers’ experiences of the issues affecting access to predominantly hospital-based services for patients with severe chronic kidney disease [3]. Key themes included difficulty in attracting and retaining skilled staff in remote areas; inadequate resources to comprehensively engage with patients to discuss treatment options and to assist them in making informed decisions regarding their health and wellbeing; poor communication between hospital and primary care providers leading to lack of coordination of care; and the need for more Aboriginal and Torres Strait Islander staff, particularly in senior decision-making roles. Overall these issues led to patients feeling poorly informed, confused and frustrated with being unable to effectively communicate their concerns [4]. The Audit and Best Practice for Chronic Disease research program is a continuous quality improvement program being undertaken in several Aboriginal health services and informed by the Wagner Chronic Care Model [5,6]. It has identified strengths and weaknesses in all Chronic Care Model domains. Key strengths included incorporation of chronic illness objectives at all management levels, enhanced community linkages, patient centred delivery systems such as transport services, gender-specific clinic spaces, and robust information technology systems. Weaknesses across all domains were predominantly related to grossly inadequate human and financial resources [7].

The work presented here forms part of the Kanyini Vascular Collaboration, a health services research program established in late 2006. The program comprises of multi-methods studies to understand barriers and enablers to high quality care, with a particular focus on vascular diseases. Studies include the Kanyini Audit, a random case record audit of eight health services, identifying prevention and management practices relating to chronic diseases [8]; the Kanyini Health Systems Assessment (the subject of this paper); the Kanyini Qualitative Study, comprising individual interviews of service providers and community members; and an intervention component comprised of a series of innovative strategies to improve the prevention and management of vascular diseases [9,10]. In this paper we analyse data from the Kanyini Health Systems Assessment with three objectives in mind: (1) to explore staff perspectives on health systems issues that impact on access to optimal primary, specialist and hospital care; (2) to determine organisational barriers and enablers to improved quality of care; and (3) to explore the relevance of these findings to the COAG NPA.

### Theoretical framework

Two theoretical concepts informed this research. ‘Candidacy’ is a concept derived from an interpretive synthesis of literature pertaining to access to health care by vulnerable groups in the United Kingdom [11]. It is defined as “the ways in which people’s eligibility for medical attention and intervention are jointly negotiated between individuals and health services” [11]. It is particularly useful in understanding health service level barriers and enablers to health care access. Three sub-themes to the theory are particularly relevant to this study. (1) *Tractability* refers to the policies, structural developments, resource allocations and interventions undertaken by services to address inequity in access to health care. (2) *Navigation and permeability of services* refers to the routes taken by people to gain a point of entry to health services with permeable services requiring little negotiation for entry and a minimal level of understanding of how the system works. Transport services, flexible appointment structures, minimal out-of-pocket expenses, and welcoming physical spaces are examples of factors that promote a more permeable service. (3) *Presentations, adjudications and offers* describes the circumstances under which people appear, and are invited or coerced into health care. The notion that health services are frequently designed to only meet the needs of an ‘ideal user’ who has a particular set of competencies and demands is particularly pertinent to this. Since the theory was first published in 2005, there have been a number of studies demonstrating its utility in both UK and non-UK settings and across a variety of health services including: aged care [12], primary mental health care [13,14], diabetes, coronary artery disease and mental illness [15], emergency department access [16], dementia care [17], and breast cancer screening [18].

Although candidacy theory has potential application to Aboriginal and Torres Strait Islander health service delivery it could be limited in accounting for the specific context of Australian health systems and the unique perspectives of Aboriginal and Torres Strait Islander people on health care constructs. The term *kanyini* is used by a number of language groups in Central Australia. It represents one of the four foundations of Aboriginal life: *Tjukurpa* (Law, Dreaming); *Walytja* (Family); *Ngurra* (Land, Country) and *Kanyini*. In essence, *kanyini* describes the principle and primacy of caring for others - an obligation to nurture, protect and care for other people, family, country and the law [19]. Myers ethnography describes Pintubi concepts of *kanyini*[20]:

*“The metaphor of ‘holding’ (kanyininpa) is rooted in a powerful experience: it derives from a linguistic expression describing how a small child is held in one’s arm against the breast (kanyirnu yampungka). The image of security, protection and nourishment is immediate. Extension of this usage characterises a wide range of relationships as variants of this mixture of authority and succour. An older woman who oversees and looks after the younger girls and women in the single women’s camp is said to ‘hold” them.”*[20]*p.212*

Franks and colleagues (1996) articulated the complexity of the concept further, highlighting that “*kanyini* is a verb which reflects a commitment, a full engagement; vitalising again and again all that went before and all that will go after” [19]. Similarly McCoy highlights how *kanyirninpa* is expressed in relationships that involve teaching and learning, and how it is viewed as an essential ingredient for social and emotional wellbeing [21]. Randall defined *kanyini* as an unconditional love and responsibility to all things [22]. In exploring *kanyini* as a potential research framework for the Kanyini Vascular Collaboration, we were mindful that it may not be appropriate for use in other settings outside of Central Australia. Although rooted in Central Australian life, there are related concepts described in health services research from other regions such as Yolngu concepts of *djāka* (caring) and *gungayun* (assisting) [23]. At the outset of forming and naming the collaboration, the use of the term *kanyin*i was discussed with participating health service partners with particular discussions directed to the non-Central Australian sites. There was strong consensus from collaborators in remote, rural and urban areas that this term and its associated concepts be explored as a part of the research program. Thus, we inductively brought together theories from the broader international literature with Aboriginal specific philosophies, resulting in a conceptual framework around candidacy and *kanyini*. In doing so we sought to explore the utility of these theories in examining the factors that influence systems of care.

## Methods

The format of the Health Systems Assessment was informed by our review of candidacy theory; concepts of *kanyini*; the findings from Improving Access to Kidney Transplants Study and the Audit and Best Practice for Chronic Disease program; and informal observations of health service systems that were made during the conduct of the Kanyini Audit [8]. Drawing on the Chronic Care Model the following four principal domains of inquiry were identified to suit local context: (1) health service governance and cultural safety; (2) workforce issues and professional standards; (3) experiences of quality improvement activities and supports; and (4) navigation of care including access to hospital and specialist services. A series of structured questions were developed around these domains. A sample of the Health Systems Assessment form and the accompanying facilitator guide is shown in Additional file 1: Attachment 1 and Additional file 2: Attachment 2.

### Setting/participants

A focus group discussion with staff members was held at seven of the eight health services which had participated in the Kanyini Audit. Due to a number of competing priorities one remote service was unable to participate. Kanyini Audit sites were selected to reflect diverse governance, funding arrangements, service activity and staffing mix [24]. According to the Australian Standard Geographical Classification [25], two services are urban, one is inner regional, two are outer regional, and two are very remote. Six services are Aboriginal Community Controlled Health Services and one is a state government funded service with a strong Indigenous management structure. Each assessment involved one staff group interview per site with three to seven participants and two research facilitators per group. We purposively sampled to ensure a diverse range of clinical, administrative and managerial staff participated in each group.

### Interview conduct

Each session was approximately two hours duration. A brief presentation and review of a site-specific feedback report on the key findings from the Kanyini Audit was provided (see sample report in Additional file 3: Attachment 3). Staff were then invited to reflect on the findings and to raise any issues they considered relevant. This served as an ‘ice-breaker’ for the remainder of the session in which an interactive discussion was held covering the questions in the Health Systems Assessment form (Additional file 1: Attachment 1 and Additional file 2: Attachment 2). Sessions were facilitated by two external research staff. Facilitators had detailed experience of working in AMSs and had a particular appreciation of local context having been involved in data collection for the Kanyini Audit. Staff were encouraged to give expansive responses to the structured questions and these were digitally recorded and professionally transcribed. Basic demographic and professional background details were collected and any relevant field notes and observations made by facilitators were also included in the analyses.

### Analysis

Interview transcripts were thematically analysed. A coding framework was iteratively developed over a series of workshops and fortnightly teleconferences. Although informed by the theories of *kanyini* and candidacy*,* this framework was inductively derived from ‘free coding’ a selection of transcripts from the Kanyini Qualitative Study and preliminary readings of the Health System Assessment group interviews. Free coding was first undertaken independently by each team member. Then, over a series of team conferences, interpretations and reflections were aired and discussed and via this iterative process a detailed coding framework evolved. This famework was then used to analyse the seven group interview transcripts from the Health Systems Assessment and the remaining semi-structured interviews in the Kanyini Qualitative Study. Coding was performed using NVivo 8 (QSR International Melb, Vic). An emphasis on researcher reflexivity influenced our analysis. Eight research team members were involved and each had unique approaches to perceiving and interpreting the data, particularly in relation to their health professional background and cultural heritage. Alongside researchers from the two coordinating research institutes were Indigenous Research Fellows employed by four of the participating AMSs. Retaining this local representation for the analysis stage brought out personal perspectives on the health service environment, interview participants and their stories. Training of team members less experienced in research and analysis was integrated into the early stages of analysis and training in use of NVivo 8 was provided.

### Ethical considerations

Five site specific human research ethics committees, including one Aboriginal committee, reviewed and approved the study protocol. These included committees constituted by the Aboriginal Health and Medical Research Council, Cairns Base Hospital, Princess Alexandra Hospital, Central Australian Human Research Ethics Committee and the Northern Territory Department of Health and Community Services and Menzies School of Health Research. Memoranda of understanding and/or partnership agreements were established between the coordinating research institutes and the respective governing bodies at each health service. These covered all components of the Kanyini Vascular Collaboration program. Health service partners and other interested stakeholders were kept informed of the study’s progress via a quarterly newsletter, website updates and annual investigators’ meetings. Project staff at the two co-ordinating institutes also had a regular presence at each AMS site, conducting on average two-monthly face-to-face visits and fortnightly telephone meetings. For several sites this regular engagement occurred over a three year period and is ongoing.

## Results

Seven Health Systems Assessments involving 37 staff were conducted between May and July 2008, soon after completion of the Kanyini Audit. Table 1 provides a profile of the services and professional categories for the participating staff. Five core themes were identified: (1) AMSs are different from private general practice; (2) AMSs are under threat; (3) a pressured workforce; (4) drivers for quality care; (5) candidacy to hospital and specialised care

**Table 1:** **Health service characteristics for the health systems assessment**^**a**^

**Service**	**Urban 1**	**Urban 2**	**Regional 1**	**Regional 2**	**Regional 3**	**Remote 1**	**Remote 2**
Service population (% regular clients)	3444 (63%)	2882 (76%)	504 (63%)	748 (76%)	11740 (70%)	780 (72%)	1100 (91%)
Indigenous governed	No	Yes	Yes	Yes	Yes	Yes	Yes
Indigenous manager	Yes	Yes	Yes	Yes	Yes	Yes	No
**Workforce total (Indigenous)**	26 (9)	42 (23)	6 (5)	9 (4)	133 (103)	7(2)	12 (6)
General Practitioners (Indigenous)	4 (1)	6	1^b^	2 (1)	8	1	1*
Registered Nurses (Indigenous)	9 (1)	1	2 (1)	1	14 (2)	4	3
Aboriginal Health Workers	2	3	0	2	17	2	2
Allied health staff	3	0	0	0	2	0	0
Chronic disease specific staff	Yes	No	No	No	Yes	Yes	No
**Systems**							
Electronic record system	Practix	MD^c^	Ferret/ MD^c^	MD^c^	MD^c^	Communi-care	Communi-care
Automated pathology	Yes	Yes	Yes	No	Yes	Yes	Yes
Disease register system	Yes	Yes	Yes	No	Yes	Yes	Yes
Home medicines review	No	No	No	Yes	No	No	No
Transport services	No	Yes	Yes	No	Yes	Yes	Yes
**On-site specialist services**							
General physician	2 weekly	2 weekly	-	monthly	weekly	2 monthly	yearly
Cardiologist	-	-	-	-	-	6 monthly	yearly
Nephrologist	-	-	-	-	-	-	-
Ophthalmologist	-	-	-	-	-	yearly	yearly
Podiatry	-	weekly	-	-	-	6 monthly	-
Dietician	daily	-	-	monthly	daily	-	3 monthly
Dentist	daily	daily	-	-	daily	2 monthly	6 monthly
**Local hospital services**							
Cardiology	Yes	Yes	No	Yes	Yes	Yes	Yes
Cardiac rehabilitation	Yes	Yes	No	Yes	Yes	No	No
Nephrologist	Yes	Yes	No	No	Yes	No	Yes
Dialysis	Yes	Yes	Yes	Yes	Yes	No	Yes

**a.** Staff participants.

Urban 1: Clinical director, GPs (x2), Chronic disease RN, AHW project officers (x2), Aboriginal liaison manager.

Urban 2: Board member, GP, AHWs (x2), RN, Finance manager.

Regional 1: Chief Executive Officer, GP, RN, Driver, Receptionist.

Regional 2: GP (x2), RN, AHW (x2), Receptionist.

Regional 3: GP, Chronic disease AHW, Cardiac Rehabilitation AHW, Programs Coordinator.

Remote 1: GP, RN (x2).

Remote 2:RN (x3).

**b.** Part-time locum only.

**c.** Medical Director.

### Theme 1: AMSs are different from private general practice

At all sites staff emphasised the unique aspects of AMS service delivery when compared with private general practice. In particular, engagement with local Aboriginal and Torres Strait Islander communities was repeatedly affirmed as the main difference. Staff viewed the type of care they provided to be comprehensive, responsive to community expectations and patient rather than business oriented. By contrast, private general practice was viewed as focussing on maximising business revenue and providing reactive rather than preventive health care. It was also felt to inadequately acknowledge the particular needs of Aboriginal and Torres Strait Islander people.

I suppose, as an Indigenous doctor, you often get (patients saying) “I’m happy to talk to you about this, but I wouldn’t really want to talk to the GP down the road about it… If it’s something to do with emotional, cultural, spiritual stuff, then that really does need to be addressed. But, you know, mainstream practices might not see it as ‘true’ medicine. (GP1, regional AMS2)

Although community linkages are known to be an important component to chronic care, the depth of community connection in AMSs goes beyond this. Even for the only non-community governed health service, staff stressed the importance of ensuring community input and that this is usually not appreciated in mainstream services.

Even though we're a mainstream health service we do work really strongly with the community. There’s nothing more important than having local people (on staff)… that liaise between the community and us… We still have that strong contact, especially with the elders… Normally mainstream health services never venture out in Indigenous health to actually work with the community and not many (patients) come to them. (Clinical director, urban AMS1)

Consistent with our theoretical understandings of *kanyini,* staff frequently commented on the obligations they felt to reach people and act in their best interests. This need to hold and nurture people was most profoundly felt by Aboriginal Health Workers (AHWs). One AHW stated that her work ‘*doesn’t just stop when we finish work*’. These obligations constitute a powerful mechanism for enhancing the candidacy of Aboriginal and Torres Strait Islander communities to health care. For AHWs there was an unconditional quality to the care provided, subtly blending the more demarcated work responsibilities with diffuse personal obligations in the community. Whilst these obligations may manifest quite differently for non-Indigenous staff a similar dedication beyond the ordinary was apparent. This duty to reach people also helps explain why health promotion constitutes a key part of service activity. Bridging clinical services with activities that develop community capacity were viewed as central to health service function.

Daniel (pseudonym), an Aboriginal project officer, works on a shared responsibility agreement with the football club.… I think that is a really good example of delivering health in a very different way and engaging the community’s strengths. Rugby league is a huge factor for a man and it shows in figures that men attending the clinic are still under represented… So this work has seen an investment of infrastructure in the community sector as well as furthering this clinic. (AHW project officer 1, urban AMS1).

In order for an AMS to ‘hold’ and nurture its community, this engagement is needed at all levels of the organisation, not just with the governing board. The employment of local Aboriginal and Torres Strait Islander staff across a variety of positions allows this holding to be adequately enacted. It affirms community linkages and the consequent legitimacy of the organisation.

Being a community controlled service you not only have it (community control) at the board level but it should be reflected in the organisational structure right through to even the groundsmen…it gives the staff themselves a sense of belonging and knowing that it is owned by the community. We all live in this community so we're a part of the organisation and we’re working for it, showing to the wider community that we are able to work at all these different levels.(AHW1, regional AMS3)

A key component to enhancing candidacy to health care is that services are easily navigated by their users. Staff from all professional backgrounds particularly commented on the availability of transport services as a key component to a navigable health service. Rather than merely an ancillary support, transport was viewed as an integral part of health care itself. Staff commented that health care standards were heavily influenced by the availability of transport and that its absence ‘*defeats the purpose of us being here*’. For the two remote services, transport was critically important. One service provided daily visits to homelands and transport to the major referral centre for acute or specialist care. This consumed substantial monetary and human resources. For the other remote site airplane transport services were especially dire with long wait times and patients having to travel alone to attend appointments. This left many feeling vulnerable when ‘*stuck*’ without family in the referral centre. For some people this impacted greatly on future decisions to seek specialist care. Thus transport is a key mechanism by which people are supported to navigate the system.

### Theme 2: AMSs under threat

Despite the primary importance of firm connections to community, several AMSs felt that their community governance structures were under threat. Many staff commented on the challenges of having to compromise community needs and expectations in order to satisfy the expectations of their principal funding body, the Office of Aboriginal and Torres Strait Islander Health. This was particularly noted in relation to complex reporting requirements. A robust governing board was considered critical to balancing these tensions.

The Board are better equipped and better able to run the health service because they're from the community, they know what the community need. To me it's a best practice approach, it's an evidence based approach because we are the community. We know what we want. We don't always have to be told “you need this, you need that”, or “ you should be doing this”… (AHW1, regional AMS3)

For the two services auspiced by Aboriginal Community Controlled Health Services from other regions, staff felt the fundamental principle of community governance was being undermined. For one service, this left many staff feeling despondent.

There’s probably to some extent a fundamental flaw in that we aren’t as community controlled as we’d like to be. We’re controlled by a different community, which just doesn’t make sense. (The local Board is) a bit of a toothless tiger unfortunately… (GP1, regional AMS2)

This volatile relationship often resulted in confusion amongst staff about who are the decision makers and what are the appropriate lines of management. At one service there was frustration that “*the bigger picture seems to get lost”* amidst a convoluted administrative structure. By contrast, staff at one large and experienced AMS considered they had a duty to support new AMSs in the region and the experience in auspicing several fledgling health services was recounted positively. These arrangements were always intended to be temporary with the view to supporting the health service to become independent. Another frequently perceived threat is that government health service providers do not consider AMS service provision to be adequate, thereby legitimising increased external involvement. In this poignant interaction between a board member (P1) and AHW (P2) both felt there was a hidden agenda to eliminate AMSs altogether, replacing them with poor quality ‘mainstream’ health care.

P1: They (government community health services) shouldn’t think that they are superior to the AMS team. That sort of an attitude, they should cut it out.

P2: That attitude will stay around for a long time until the boss of this organisation says something to them.

P1:They say that we need their services but that doesn’t mean they should come and tell us to do this, do this, do this… They try to bung low grade services onto us… If we look a little bit further down the track, say five or ten years, there won’t be any more AMSs. They will have become mainstream services.

P2: That’s a plan of the minister… low grade services.

(Board Member, AHW1, Urban AMS2)

This perception appeared to be driven by a long history of negative interactions between government officials and the AMS and a failure to agree on collaborative models of service provision. In contrast to such accounts of being consumed by the mainstream, there were examples of successful partnerships between AMSs, government and other non-government agencies. One Aboriginal Health Worker argued for a ‘co-operative self-determination’ where government agencies practise non-interference whilst still maintaining support to AMSs to self-direct the delivery of health care. An example of this in practice at one site involved the establishment of an inter-agency forum over 14 years ago which has allowed government and non-government organisations (NGOs) to have equal representation and to collaborate successfully on several projects.

### Theme 3: A pressured workforce

Three key workforce-related issues were raised by staff: (1) a lack of staff, (2) AHW roles and support, and (3) access to professional development.

#### Lack of staff

Several people considered that chronic staff shortages curtailed the quality of care they provided. This was the major contributor to staff burn out. In particular, insufficient staff to meet acute care needs was considered a critical barrier to developing sustainable chronic disease services.

In the past, patients would have a preventive health check but this has stopped because there's been an influx in acute care….That leaves the doctors no time to manage chronic needs and help patients to self-manage… So I think we need to look at how are we as an organisation going to tackle acute care… This would then have an impact on chronic disease because a lot of the acute problems are manifesting as chronic disease later… (GP1, regional AMS3)

The Kanyini Audit found that around one in three routinely attending adults were at high risk of vascular diseases. This highlights that patient care cannot be easily dichotomised into acute and chronic care. Chronic care is likely to be everyone’s business, whether in the specialised or general clinic setting. The corollary to this is that adequate resources are essential in order to provide comprehensive chronic care services to routinely attending patients, regardless of the reason for the encounter.

#### Aboriginal Health Workers roles and support

In candidacy theory, tractable organisations have policies that are specifically geared toward increasing access for vulnerable populations. AHWs appear to be one of the key mechanisms to making AMSs more tractable. Their ability to expand the type of care provided and their unique skills in engaging with community members were viewed as a critical adjunct to conventional medical services. This was not merely related to their identification as an Aboriginal and/or Torres Strait Islander person. One Aboriginal GP commented that an AHW “*is a key person that community members will feel more comfortable talking to about stuff than even with me”*. Via the AHW he benefited from an enhanced flow of information and feedback from the community to better inform his care provision. The diverse roles fulfilled by AHWs were evident in this study. In several sites a more traditional AMS model operates in which the AHW provides the first point of contact for triage and health screening. Brokerage roles such as making health information more accessible are a key part of this role.

Doctors tend to talk big words and a lot of community people don’t understand that. So I'll break it down into our jargon and I put it straight to them… (AHW2, urban AMS2)

Juxtaposed with these traditional clinical and brokerage roles is an increasing emphasis on health promotion and community development roles. For one project officer the delivery of ‘*strengths based health promotion*’ and community empowerment strategies were central to her role.

Where I feel most comfortable is around community-based health promotion and delivering health from an Indigenous perspective of health. So not just thinking about food and exercising but thinking about community well-being. (AHW project officer 1, urban AMS1)

Although these roles were acknowledged at most sites as critical components to health care, at one remote service, non-Indigenous clinic staff considered their AHW staff to have only a nominal presence, suggesting they served no useful function at all, acting as ‘pin-ups’ rather than as providers or health care. Further they considered family obligations and kinship relations to be a barrier to care.

There’s a lot of issues because for both of our health workers there’s family groups that they won’t go near… I just think that being a health worker here is a position that doesn’t have any credence or respect in the community… Ideally the health worker here would be someone who’s Indigenous and knowledgeable with the people, but who’s not from here, who is impartial to all the different groups of people here. (RN1, remote AMS1)

Such comments stand in stark contrast to the preceding ones in which AHWs play an expansive role as the nexus between service and community, broker of services, coordinator of health promotion activities and embodiment of a community governance model. They emphasise a more narrow view of AHWs as medical assistants. These apparent weaknesses of over-familiarity with the community can constitute a substantial asset to the health service if supported appropriately. The level of organisational support for these roles is therefore a critical factor in maximising the contribution of the AHW workforce to improved care.

#### Workplace orientation and professional development support

Although access to professional development opportunities seemed well supported, few sites had any formal orientation to prepare staff for working in AMSs. Only the state government service has obligatory cultural awareness training workshops for new staff. Many commented, however, that this course was tokenistic and poorly prepared staff for the realities of their work on the ground. Despite this limited orientation, staff commented on a wide variety of informal measures through which they gained experience and support to carry out their job roles. In particular, senior Aboriginal and Torres Strait Islander staff, community members and elders were highly valued as a source of guidance on professional conduct. Fulfilling such roles poses challenges, however, for these senior people. In one AMS this meant that the senior doctor was managing a unique and extraordinarily high workload. His multiple perspectives as an Aboriginal person, respected manager and medically qualified health professional resulted in him supporting a variety of staff on a breadth of issues. These demands were noted by one staff member:

“It’s very refreshing and very exciting having one of the other doctors who is Indigenous… Everybody wanted him because he’s confident, affable and smart… Although it was fantastic having him in that role, it was also very difficult for him to actually perform it because he had so many other responsibilities” (GP1, Regional AMS3)

### Theme 4: Drivers for quality care

Three components of the Chronic Care Model were most talked about in relation to effective quality improvement (QI) strategies. These were related to organisational influence (especially leadership), information systems, and delivery system design (especially care planning and follow-up)

#### Organisational influence and leadership

Interest and capacity at the highest levels of management appear key to establishing satisfactory QI systems in AMSs. At one service, three senior positions (executive officer, medical director and a dedicated systems manager) took responsibility for quality improvement activities. Of particular importance was the link between the medical director and non-clinical managers. For many years this productive relationship resulted in a growth of quality improvement activities. In more recent times, however, with the medical director position not filled for some years and high turnover with the systems manager position, QI activities have diminished in prominence. At another site, staff felt that managers were out of touch with their needs and made decisions that were ill-informed or cost-driven rather that patient care driven.

To provide good chronic disease management you need great systems and at the moment we've had problems with that…. From experience, systems are always forced upon us by higher management… systems we don't want. (Clinical director, urban AMS1)

#### Delivery systems and care planning

Sufficient numbers of adequately trained staff for quality improvement activities was extensively discussed. There were a variety of approaches to how staff should take responsibility for recall systems and chronic care planning. In two services, registered nurses had the primary responsibility to manage recall systems whilst at another service, AHW program coordinators managed disease-specific programs such as diabetes and cardiac rehabilitation. This contrasts with another service’s experience where chronic care coordination was provided as a part of a joint state and federal funded initiative conducted as a collaboration between three AMSs and several regional health providers. Under this program externally funded ‘care units’, based at the AMS, initiated health assessments and care plans. Additional funding was also available to assist patients to access specialist services under this scheme. Whilst care coordination has been trialled successfully elsewhere in Australia, the AMS staff we interviewed felt that the program was overly driven by those stakeholders who were external to the organisation. These external staff were considered to be overly focused on data collection for statistical reports and less committed to actual coordination of care. There were mixed views about the role of rebated health assessments and care plans as a component to chronic care delivery systems. In Australia these items are publicly funded through Medicare, the federal government’s universal health care system which provides Australian residents with subsidised health care costs for services provided by a health professional. For one service an upfront investment in nursing staff to manage Medicare rebated chronic disease services proved to be a successful business strategy as the income generated could offset the funding allocated for these positions. At another service, however, the chief executive officer cautioned that when this became the end rather than a means to quality patient care it could compromise the integrity of the service.

You can spend all your time chasing Medicare dollars… you can do health assessments just for the sake of doing health assessments and not actually help the patients…. It’s not necessarily the great pot of gold…it’s not going to solve all your problems… And chasing a handful of dollars, sometimes you don’t pursue the right directions, and your directions should be primarily improving the health of the community that you’re working with. (CEO, regional AMS1)

#### Information Computer Technology/ Information Management (ICT/IM)

Many staff felt their ICT/IM infrastructure was sub-standard. This constitutes a major barrier to supporting a culture of quality improvement in chronic care. The most common and serious concern was that systems had frequent outages and that support services were inadequate to troubleshoot problems when they arose. Staff were concerned about potential loss of important information and disruption to clinical care. Dissatisfaction with electronic health record systems was equally important. Of the various software systems used there appeared to be strengths and weaknesses with each of them with no one system being an ideal fit.

I would love to marry Ferret, Communicare and Medical Director all into one… They all have something that's so good and then there's a part of them that’s so crap… We all need different bits out of it. so the doctors need the clinical side, health workers need the management side and the Board and management need data for their reports, for funding purposes. But one program never gives all of it… I think the person who comes up with this program is going to be a national icon! (AHW2, regional AMS3)

Equally important was the lack of staff training to fully reap the benefits of whichever system was being used. Staff felt that even after several years of use they were still discovering new things. Thus despite the varied QI strategies in place, a common theme was the need for high level leadership with a strong investment in nursing and/or AHW staff. Adequate information systems are desperately needed with barriers operating at the level of users (poor training and support), the environment (poor infrastructure) and unsuitable software systems.

### Theme 5: Candidacy to hospital and specialised care

Another key Chronic Care Model component to good delivery systems design is the degree of coordination between primary care and specialist services. Staff identified several factors that influenced people’s candidacy to care. These related to poor hospital communication, experiences of discrimination and difficulty in navigating specialist services. These factors substantially compromise the ability of health systems to hold, nurture and protect their users. There were mixed accounts regarding satisfaction with the care provided by hospitals. In the urban and larger regional centres staff were generally satisfied with services provided and the level of communication. At the smaller rural and remote sites, however, AMS staff felt that communication processes were highly variable and dependent on the conscientiousness of staff working on a particular roster. At one remote site, staff were frequently not aware of an episode of hospital care until the patient presented to the AMS for medications. At the other extreme, patient information was often faxed to an AMS who was not the regular care provider and this would create burdensome work ensuring that other health services were notified. Experiences of discrimination in the hospital and specialist care systems were pervasive in all settings. There were frequent stories of hospital staff displaying hostile attitudes to patients. These were often fuelled by stereotypical assumptions about Aboriginal people.

We had a young Aboriginal fella, he went to the hospital and the nurse asked him “when was the last time you had a bath?” … (The person then walked out). And I heard about this young fella over the weekend and Monday morning, we went looking for him. We brought him in to see the doctor. It turned out he had an abscess on his lung which was really serious and he had to be hospitalised straight away…(AHW1, regional AMS1)

The volume of discriminatory accounts experienced by patients means that AMS staff are frequently intervening on the patient’s behalf to restore candidacy. For one urban AMS GP, this involved *‘a lot of advocating… ringing and cutting through the crap, the resistance and the verbal ‘ rolling of the eyes’ and just keeping on pushing until the appointment happens’.* Access to specialist services in remote areas was a substantial problem in the two services we interviewed. Consistent with the ideal user concept, remote area specialist services appear to struggle with the need to adapt systems for circumstances that are quite different to those in the city.

One of the issues is that people at the end of the line are so busy.that they don’t actually think, “gee this person comes from a 1000 km away, so we actually have to think differently”. Yes, we need to do what we would do for everybody else but hey, we can’t send them back and get them back next week (GP1, remote AMS1).

In one service this led to devastating consequences where a patient with suspected coronary heart disease was asked to travel back to her community to await coronary angiography at a major referral centre. During this waiting period she had a fatal heart attack. In regional and urban areas specialist availability was also a challenge. AMSs in those areas are continually cultivating networks of specialists whom they know are favourably disposed toward providing care to Aboriginal and Torres Strait Islander people. Eliminating financial barriers through the availability of bulk-billing specialists is a key consideration for determining suitable specialists for referrals. Although these specialist networks tend to develop in an *ad hoc* manner, there were several examples of highly successful partnerships that are improving access to specialised services. The primary feature to these partnerships was the enhancement of on-site service provision. At one AMS a cardiac rehabilitation service was established through formal agreements with the hospital. Central to the program’s early success has been management by an AHW who has himself experienced a heart attack. In this way a specialist service can be delivered by trusted local staff on site with access to the full range of other AMS services such as transport, dental care, social and emotional well-being services and general primary care. In line with these initiatives, some services have engaged in purposeful capacity building to allow GPs at AMSs to provide services usually performed by specialists, including retinal screening programs and intensive insulin management.

## Discussion

This analysis of AMS health systems provides a useful snapshot of staff perceptions of barriers and enablers to health care access and quality for Aboriginal and Torres Strait Islander people. Although our focus was on identifying better systems of care for chronic diseases, the findings that emerged appear to be indicative of much broader health systems issues. Candidacy and its sub-constructs provide a useful frame for describing and understanding health care access issues in this study. We found that tractable and navigable health services have good governance structures, sound leadership, systems that welcome the ‘non-ideal user’, good patient transport systems, and a well-supported workforce. Similar findings on navigation barriers and enablers have been noted in the international Indigenous health literature, particularly availability of transport [26-30], minimal or no out-of-pocket costs for attendance and treatments [27,31-34], welcoming physical spaces [30,34-36] and the ability of a health service to serve as a social and community space [37]. The relevance of *kanyini* is different but complementary to candidacy. It helps to clarify the distinguishing features of health care provision in AMS settings when compared with mainstream government and private services. The findings highlight multiple strategies taken by AMSs to ‘hold’ people from childhood to old age. These include robust community governance, community representation on staff and linkages with other community organisations, strengths based health promotion activities, and most notably the extra-ordinary efforts to reach people who may not otherwise be able to access health care. The degree to which a person, family or community feels held may be a fundamental driver of whether care is viewed as ‘proper’ [21]. Several findings from this study have implications for the implementation of policy initiatives under the Council of Australian Government National Partnership Agreement on Closing the Gap in Indigenous health outcomes (COAG NPA). These are related to AMS sector support and staffing initiatives, discrimination in hospital care, increasing candidacy to specialist care, and overcoming health service systemic barriers.

### AMS sector support and staffing

The duty felt by AMS staff to properly ‘hold’ people is undermined by a substantial fear about the viability of the AMS sector. Despite the AMS sector having a stronger national presence than ever before, staff remain suspicious of government intentions. The COAG NPA has had mixed responses with many concerned that these initiatives are geared toward enhancing Aboriginal and Torres Strait Islander people’s access to private general practice services, and that they are neglecting the substantial role played by this sector [38]. Although our Health Systems Assessments were conducted prior to implementation of the COAG NPA, it is quite likely that these new initiatives would further compound rather than allay the feeling of being under threat. The findings also shed new understanding on why the employment and support of Aboriginal and Torres Strait Islander staff is a critical component in promoting candidacy to health care. Staff highlighted that Aboriginal Health Workers are one of the essential elements to ensuring that people are properly held by their health services. Other studies from North America and New Zealand have similarly highlighted the broad roles played by Indigenous health workers including working as clinicians and health promoters [39], brokering better delivery of health information [40,41], and fulfilling responsibilities to patients as friends and family whilst maintaining professionalism and avoiding nepotism [37,42-45]. The COAG NPA has invested in several hundred new Aboriginal and Torres Strait Islander positions including tobacco workers, lifestyle workers, outreach workers and self-management workers [1]. Whilst such a large workforce commitment may be a sound investment there are cautionary aspects to this policy. The ability of an AMS to ‘hold’ its community is equally applicable to its staff, especially its Aboriginal and Torres Strait Islander staff. Given a large proportion of these new workforce positions will be based in mainstream primary health care organisations (known in Australia as Medicare Locals or formerly Divisions of General Practice), there is potential to shift existing AHWs away from the AMS sector into isolated organisational contexts. Further, there are important professional development needs that must be addressed. Despite a national Aboriginal and Torres Strait Islander workforce strategy being developed in 2002 [46], progress on implementation has been slow and barriers to improving workforce standards remain [47,48]. The recently created National Aboriginal and Torres Strait Islander Health Worker Association may help to address this with new national registration and accreditation standards. It is hoped that this agency will provide professional development opportunities that are flexibly delivered and recognise the diverse roles played by AHWs. Institutional supports such as these are an important mechanism to better ‘holding’ this workforce.

### Discrimination in hospital systems

Whilst there were relatively minor frustrations about hospital systems (especially communication processes), the most concerning issue was the repeated accounts of perceived discrimination experienced by patients. Such discriminatory attitudes emphasise that Aboriginal and Torres Strait Islander people may be viewed *a priori* as ‘non-ideal’ users and treated in a hostile manner by the hospital system. Candidacy theory describes how this makes the hospital system highly intractable and helps explain the circumstances culminating in ‘leaving hospital against medical advice’ and ‘non-compliance’ with medical instruction [49,50]. These findings support those of others in which racism in health care is highly prevalent for Aboriginal people, impacting on personal health and eliciting a range of constructive and destructive coping strategies to manage its effects [51]. Dealing with these negative experiences appears to be a regular component of AMS health professionals’ work. A specified objective of the COAG NPA is to ‘fix the gaps and improve the patient journey’. One performance benchmark for this objective is that state and territory government implement strategies to improve cultural security and practice within public hospitals. Despite this being explicitly stated there are few implementation initiatives that appear to be addressing this. Discussion of institutional and interpersonal discrimination in hospitals is beyond the scope of this paper, but it is likely that conventional cultural awareness training workshops do little to address such a complex and highly pervasive issue. Some potentially instructive alternative strategies include anti-racism training [52] and the development of tools that critically examine notions of culture, race and oppression [53-55]. At an institutional level, New Zealand Māori advocates have called for organisational audits for compliance with Treaty of Waitangi principles [56]. In addition, several New Zealand district health boards have policies outlining *tikanga* best practice guidelines for respecting Māori principles in relation to hospital care [57].

### Improving specialist service access

On-site specialist outreach clinics appear to be a beneficial strategy to enhance the proper holding of people. Our findings complement those found in the evaluation of the Northern Territory Specialist Outreach Program [58]. Although such services are likely to meet the chronic care needs of a minority of clients, there are delivery system benefits beyond making services more permeable and navigable. On-site services foster increased trust and sound collaborating relationships between AMSs, government and private agencies. They enhance the ability of AMSs to hold clients in the system by better coordinating primary and specialist service delivery. The use of adequately supported and trained AHW coordinators can impart a strong nurturing component to these services. At a systems level, on-site services can enhance professional development opportunities for local staff (eg. via case conferences, journal clubs, and training in technical procedures), which can lead to a sustainable enhancement to workforce capacity. The federally funded Medical Specialist Outreach Assistance Program complements state and territory outreach specialist programs to rural and remote communities but specialist service support schemes remain piecemeal and fall considerably short of being systematic and comprehensive. Within the COAG NPA, increased funding is being provided to expand the program [59]. A key component of this expansion is to foster the development of multidisciplinary teams, but questions remain whether these initiatives are adequately resourced to meet demand. Our study findings support this policy decision and, if adequately financed, there is the potential to make an important contribution to improving the navigation of specialist care.

### Overcoming health service systems barriers

In addition to the importance of *kanyini* and candidacy, we identified a number of systems barriers to uptake of federal policy initiatives. The use of Medicare incentives to promote better systems of chronic care is of particular relevance to the COAG NPA. Financial incentives are being provided to AMSs and private general practices for registering and providing a minimum number of Medicare services to Aboriginal and Torres Strait Islander people with or at higher risk of a chronic disease [1]. Low uptake of these Medicare items has been well documented in both the Kanyini Audit and elsewhere [8,60]. We identified several system issues in this study that might contribute to this, especially poor information management and inadequate staff resources. Perhaps more important, however, were the mixed views of the value of these Medicare items on patient care. Whilst the larger sites felt incentives could assist in providing comprehensive care and additional business revenue, at the smaller sites they tended to be viewed as a distraction from rather than a promoter of good health care. The National Aboriginal Community Controlled Health Organisation has voiced similar concerns that the use of Medicare incentives in the COAG NPA may create an ‘inverse care’ situation where those least in need of care will be more likely to receive these Medicare services and *health* services with the least capacity and those patients with more complex care needs may miss out [38]. Close monitoring is needed to identify if such uptake patterns emerge. Novel funding models to enhance Aboriginal and Torres Strait Islander people’s access to services have been proposed by the Australian National Hospitals and Health Reform Commission [61]. Similar to the Australian Department of Veteran Affairs model, eligible patients would receive universal entitlements to particular services. This would allow the patient to be the arbiter of which services to access and care providers would be able to claim benefits from this funding authority. Given the substantial restriction in choice of specialist provider and the accompanying financial and transport barriers discussed in this study, innovative models could address navigational barriers in the health system. Despite its recommendation, it does not feature at all in current government health reform discussions. The ICT/IM barriers we encountered are highly consistent with a major Office of Aboriginal and Torres Strait Islander Health review of health service views on reporting requirements [62]. The Aboriginal Health and Medical Research Council has also conducted a series of organisational audits on ICT/IM capacity in NSW AMSs [63]. This review found a considerable shortfall in budget allocation toward ICT/IM systems, low levels of ICT/IM governance, and poor computer literacy amongst staff members. These issues are again consistent with our study findings and warrant urgent attention. The COAG NPA includes a component for web-based reporting and monitoring tools for Office of Aboriginal and Torres Strait Islander Health funded organisations [1], but there are few specific initiatives to address infrastructure barriers and staff support.

### The potential role of kanyini and candidacy in future policy development

Incorporating *kanyini* and candidacy theories into a coherent health policy framework should not be viewed as an academic exercise. McCoy uses *kanyini* to explain the excellent educational outcomes that were achieved in one central Australian school [21]. The manner in which this institution ‘held’ people, honouring its obligations to nurture and ‘grow’ its students is compatible with a systems oriented approach. The 2008 Close the Gap National Indigenous Health Equality Targets, proposed by a coalition of over 40 leading non-government agencies, are well aligned to the principles of *kanyini* and candidacy [64]. There are five interlocking sets of targets with a focus on: (1) partnership with Aboriginal and Torres Strait Islander people in the design, delivery and control of health services to optimise access; (2) health issues responsible for the life expectancy and child mortality gaps; (3) health services required to address those health issues with an emphasis on capacity building and optimal access to support programs; (4) health service infrastructure investment especially in workforce and capital works; and (5) targets associated with upstream social determinants [64]. The ‘Close the Gap’ Coalition emphasised that all five sets of targets are required to ensure progress. Such targets could be viewed as practical implementation strategies that are highly consistent with *kanyini* and candidacy theory.

## Conclusions

Forthcoming analyses of the semi-structured interview data in the Kanyini Qualitative Study will assist in determining the consistency of the findings presented here. Nevertheless, we conclude that the frameworks of *kanyini* and candidacy are useful theoretical foundations. Although further work is clearly needed, they hold promise for providing a policy framework for enhancing AMS sector contribution to health improvement and may be of value in other health service contexts that provide care for underserved, marginalised or vulnerable populations. With federal and state governments embarking on major health reforms for all Australians, large scale qualitative work of this nature can play a key role in determining strategies that will lead to better systems of care.

## Abbreviations

**AHW**: Aboriginal Health Worker; **AMS**: Aboriginal Medical Services; **COAG NPA**: Council of Australian Governments National Partnership Agreement; **GP**: General Practitioner; **ICT**: Information computer technology; **IM**: Information management; **QI**: Quality Improvement; **RN**: Registered Nurse.

## Competing interests

The authors declare that they have no competing interests.

## Author contributions

AB, MH, DP, AC designed the study. MH, DP, BR, AB were involved in collection of the data. DP, BR, AB, AC contributed to the development of the coding framework. AT, IR, NH, provided frequent strategic advice for all Kanyini studies and contributed to the policy implications of the study findings. All authors critically reviewed the manuscript, contributed to amendments and approved the final version. All authors read and approved the final manuscript.

## Pre-publication history

The pre-publication history for this paper can be accessed here:

http://www.biomedcentral.com/1472-6963/12/369/prepub

## Supplementary Material

Additional file 1: Attachment 1 Health Systems Assessment form. Click here for file

Additional file 2: Attachment 2 Health Systems Assessment facilitators guide. Click here for file

Additional file 3: Attachment 3 Sample Kanyini Audit feedback report. Click here for file
