# Supplementary material for: Building better systems of care for Aboriginal and Torres Strait Islander people: findings from the Kanyini health systems assessment
Source: BMC Health Serv Res. 2012 Oct 28;12:369. doi: 10.1186/1472-6963-12-369 (PMC3529689; doi:10.1186/1472-6963-12-369)
Supplement: Additional file 1: Attachment 1 — Health Systems Assessment form. [file 1472-6963-12-369-S1.pdf]

Attachment 1: Health Systems Assessment form

Kanyini Health Systems Assessment Form

AMS code: ☐☐☐☐ **Date of interview:** **Interviewers:**

Focus group members:

________________________ ________________________
________________________ ________________________
________________________ ________________________
________________________ ________________________

Service characteristics

Estimated total service population:<br><br>Estimated number regular clients:<br><br>Estimated number visitors/ irregular clients:<br><br>Total budget:<br><br>% budget allocated for chronic disease:	Total staff: Number Indigenous:<br><br>GPs: Number Indigenous<br>RNs: Number Indigenous<br>Allied Health staff: Number Indigenous<br>Male AHWs:<br>Female AHW:

Community ownership and accessibility- Can you describe how the health service involves the community in health care delivery?

Indigenous governing board:

Indigenous manager:

Orientation to community provided to new staff:

Client transport services available:

Cultural safety- Can you describe how the health service approaches cultural issues in health care?

Written cultural protocols available	Y	N
Cultural leave provided	Y	N
Use /support of Ngangkari/ traditional healing	Y	N
Funded Ngangkari/ traditional healing services	Y	N
Availability of separate male and female spaces	Y	N

Quality of care- Can you describe the quality improvement strategies that you have or have had in place?

Routine quality improvement activities conducted	Y	N
Dedicates quality Improvement staff	Y	N
Chronic disease coordinator	Y	N
Clinical service orientation	Y	N
Professional development support	Y	N
Guidelines for chronic disease management:	Y	N
Guideline training provided to staff	Y	N

Models of care- Can you describe any systems in place to support chronic care?

Outreach clinics conducted	Y N	Computerised record system	Y N
Chronic disease prevention programs	Y N	Automated pathology available	Y N
Chronic disease case management	Y N	Routine population data	Y N
Onsite pharmacist	Y N	Recall system in place	Y N
Access to Section 100	Y N	Chronic disease register available	Y N
Home medicines review available	Y N		
Adherence monitoring available	Y N		

Hospital care- How adequate is the continuity of care with the hospital system?

Hospital Liaison staff available Y N

Care planning includes hospital staff Y N

Quality of communication:

Admission notification	☐	(score 1-10)
Outpatient department/ Specialist	☐	(score 1-10)
Discharge notification	☐	(score 1-10)

Consistency in management between hospital and health service: ☐ (score 1-10)

Accessibility of outpatient department/ hospital specialists: ☐ (score 1-10)

Regional Services- What access do you have to the following regional specialist services?

Cardiology Services

Cardiologist available	Y N				
AMS clinics	Y N	How often? ______________			
Hospital outpatient clinics	Y N	How often? ______________			
Cardiac rehabilitation services available	Y N	local ☐	regional ☐	distant ☐
Echocardiography service available `	Y N	local ☐	regional ☐	distant ☐
Stress tests available	Y N	local ☐	regional ☐	distant ☐
Angiography services available	Y N	local ☐	regional ☐	distant ☐

Renal Services

Nephrologist available	Y N			
AMS clinics	Y N	How often? ______________		
Hospital outpatient clinics	Y N	How often? ______________		
Dialysis services available	Y N	local ☐	regional ☐	distant ☐
Transplant services available	Y N	local ☐	regional ☐	distant ☐

Diabetes Services based at the AMS

Physician	Y N	How often? ______________
Ophthalmology	Y N	How often? ______________
Podiatry	Y N	How often? ______________
Dietician	Y N	How often? ______________
