# Supplementary material for: Building better systems of care for Aboriginal and Torres Strait Islander people: findings from the Kanyini health systems assessment
Source: BMC Health Serv Res. 2012 Oct 28;12:369. doi: 10.1186/1472-6963-12-369 (PMC3529689; doi:10.1186/1472-6963-12-369)
Supplement: Additional file 2: Attachment 2 — Health Systems Assessment facilitators guide. [file 1472-6963-12-369-S2.pdf]

Kanyini Systems Assessment Tool – Facilitators' Guide

ACCHS code
Aboriginal Community Controlled Health Service (ACCHS) identifier, unique number allocated to each ACCHS involved in the research project. Numerical 4 digit identifier

Date of Interview
Record date Systems Assessment interview conducted. [DD/MM/YY]

Interviewer
Record the initials of the individual conducting the Systems Assessment interview.

Focus group members
Record the initials of each individual participating in the Systems Assessment interview.

Service characteristics

Estimated service population
Record the total number of clients registered as attendees to the health service.

Estimated number of regular clients
Record the number of regular clients attending the health service (who routinely receive PHC at the health service).

Estimated number of visitors/ irregular clients
Record the number of visiting clients that attend the health service (A visitor is someone who does not live within the catchment area, but that has previously attended the health service).

Total operating budget
Record the total operating budget of the health service. NB: Answering this question is optional

Proportion for CD
Record the proportion of the budget that is specifically identified for the delivery of chronic disease care or programs. NB: Answering this question is optional

Community ownership and accessibility

Indigenous governing board
Is the ACCHS governed by a completely or majority indigenous governing board?

Indigenous manager
Is the Manager (or 'CEO', 'Administrator', 'Director' etc) of the health service indigenous?

Community orientation
Does the health service provide an orientation to the community for staff on commencement? What form does this orientation take?

Client transport service
Does the health service provide a transport service to its clients to attend the health service and/or other services/regional hospital? [Can you describe what transport is provided for?]

Total staff
Record the total number of staff employed by the health service (including clinical, administration, and ancillary staff)

General Practitioners
Record the number of GPs currently employed within the health service. Record the number of GPs who are indigenous.

Registered Nurses
Record the number of RNs currently employed within the health service. Record the number of RNs who are indigenous.

Male Aboriginal Health Workers
Record the number of male AHWs currently employed within the health service.

Female Aboriginal Health Workers
Record the number of female AHWs currently employed within the health service.

Allied Health staff
Record the number of Allied Health persons currently employed. List each Allied Health position(s).

Cultural congruence

Cultural Awareness Program
Do health service staff undertake cultural awareness training programs when commencing work at the service?

Cultural leave provision
Does the health service have provision for indigenous staff to take cultural leave for funerals etc.?

Use/support of Ngangkari/ tranditonal healing
Does the health service use and support the use of Ngangkari/Traditional Healers?

Funded Ngangkari/ traditional healing services
Are Ngangkari/Traditional Healers paid by the health service for consultations?

Separate Men's and Women's space
Does the health service have designated women's/ men's areas within the service?

Quality of care

Routine quality improvement activities conducted
Does the health service have a systematic Quality Improvement (QI) process in place? Can you describe the QI initiatives that are in place?

Dedicated quality improvement staff
Does the health service have an identified position responsible for developing, maintaining and coordinating Quality Improvement processes within the health service?

Chronic disease coordinator
Does the health service have an identified position responsible for coordinating chronic disease care? What are the responsibilities of this position?

Clinical service orientation
Does the health service have a formal clinical orientation programme for new clinical staff on commencement of employment? Can you describe what this programme consists of?

Professional development support
Does the health service encourage and provide support for staff to attend PD activities?
Does the health service have a formal Professional Development programme for staff? Can you describe what this programme consists of?

Guidelines for chronic disease management
Does the health service have accessible, user friendly guidelines available and encouraged/mandated for use by all practitioners with respect to the identification and management of chronic disease? [E.g. CARPA, NHFA, CARI, NHMRC].

Guideline training provided to staff

Does the health service provide training to staff, in the use of these guidelines? Can you describe what the training consists of?

Models of care

Outreach clinics
Does the health service provide outreach primary care services? Can you describe these services?

Chronic disease case management
Does the health service use a case management model for clients with an identified CD? Can you describe the model used?
Case management refers to planning, coordinating, managing and reviewing the care of an individual patient. This may involve, assigning each person a 'case manager' who is responsible for assessing patients' needs; developing a care plan, arranging suitable care; monitoring the quality of care; and maintaining contact with the patient and their family.

Chronic disease prevention programmes
Does the health service have any chronic disease prevention initiatives in place? Can you describe the initiatives that are in place?

Onsite Pharmacist
Is there an onsite pharmacist?

Access to Section 100
Does the health service have access to Section 100 arrangements for the supply of pharmaceutical goods?

Home medicines review process
Is there a process in place for conducting Home Medication Reviews and claiming the available Medicare rebate?

Monitoring adherence
Is there a process for monitoring adherence? Describe

Computerised record system
Is there a computerised Patient Information and Recall System (PIRS) in use?

Automated pathology
Is there an automated system for receiving pathology results?

Routine population data
Is there a formalised mechanism for providing regular population health data to the community and health service staff?

Recall system
Is there a client recall system in place that supports the delivery of or 'recalls' individuals to receive scheduled services?

Chronic disease register
Is there a register of all clients with chronic disease?

Hospital care

Hospital Liaison
Record if the health service employs a hospital liaison officer.

Care planning includes hospital staff
Record if hospital staff are included in the development of Team Care Arrangements [EPC 723 or equivalent].

Communication, Accessibility and Consistency of Hospital Based Services.
The following questions seek to build consensus among focus group participants, by considering the usual process and barriers to care and ease of communication between primary care staff and hospital staff when arranging [or attempting to arrange] admission, referral, assessment and management by specialists within hospitals, and that occurs following a patients' discharge from hospital.

**Admission:** Describe the ease of communicating with and accessibility of hospital services when attempting to arrange the admission of a patient to hospital for assessment or management?	1<br>Extremely difficult to communicate and arrange admission	2<br>Significant barriers to admission exist	3<br>Access to admission is often, but not always, difficult	4<br>Admission and communication is good, but occasional difficulties exist	5<br>Well developed systems and/or relationships that ease admission
**OPD/Specialist**: How would you describe the ease of communicating with and accessibility of hospital outpatient or specialist services?	1<br>Extremely difficult to arrange OPD appointments with little or no communication afterwards	2<br>Significant barriers to OPD appointments & communicating outcomes exist	3<br>Access to OPD appointments is fair with reasonable communication	4<br>Arranging OPD appointments is easy with good communication, but occasional difficulties exist	5<br>Well developed systems that ensure appointments/ communication of results
**Discharge**: How would you describe the flow of information, patient results and care requirements following a hospital admission?	1<br>The PHC service is rarely or never contacted by hospital staff prior to discharge and discharge summaries rarely are forwarded.	2<br>Significant barriers to communicating between hospital staff and PHC exist	3<br>Discharge summaries, care plans and results are often, but not always communicated to PHC staff	4<br>Communication at the time of discharge is good, but occasional difficulties exist	5<br>Well developed systems and/or relationships that ensure the flow of information following patient discharge.
Consistency in management between hospital and PHC: *To what degree are management practices, medications and follow-up similar between hospital and PHC services with relation to CD care?*	1<br>Minimal or no consistency	2<br>Care plans and discharge medications are frequently changed to fit with local EB guidelines	3<br>Consistency is fair with modifications made some of the time.	4<br>Consistency is good, but occasional modifications must be made	5<br>The care of patients with CD is consistent between Hospital and PHC

Regional Services

Cardiologist services
Does a cardiologist visit the health service to provide clinical care? If so, how often?
Is there a cardiologist at the hospital accessed by health service clients or referred to by the health service?

Cardiac Rehabilitation services
Do health services clients have access to cardiac rehabilitation services or programmes? If so, are they run by the health service or external agencies/providers?

Echocardiography service
Are echocardiography services available for health service clients? [Local, Regional, Distant Tertiary Referral]. If so, are there any difficulties for clients accessing them?

Stress tests available
Are Exercise Stress Testing services available for health service clients? [Local, Regional, Distant Tertiary Referral].
If so, are there any difficulties for clients accessing them?

Angiography services
Are angiography services available for health service clients? [Local, Regional, Distant Tertiary Referral].
If so, are there any difficulties for clients accessing them?

Renal services
Does a Nephrologist visit the health service to provide clinical care? If so, how often?
Is there a nephrologist at the hospital accessed by health service clients or referred to by the health service?

Dialysis
Are renal dialysis services available for health service clients? [Local, Regional, Distant Tertiary Referral].
If so, are there any difficulties for clients accessing them?

Transplant
Are kidney transplant services available for health service clients? [Local, Regional, Distant Tertiary Referral].
If so, are there any difficulties for clients accessing them?

Diabetes services at the AMS
Does a physician visit the health service to provide clinical care? If so, how often?

Ophthalmologist
Does an ophthalmologist visit the health service to provide clinical care? If so, how often?

Podiatry
Does a podiatrist visit the health service? If so, how often?

Dietician
Does a dietician visit the health service? If so, how often?

Dentist
Does a dentist visit the health service? If so, how often?
