# Supplementary material for: Building better systems of care for Aboriginal and Torres Strait Islander people: findings from the Kanyini health systems assessment
Source: BMC Health Serv Res. 2012 Oct 28;12:369. doi: 10.1186/1472-6963-12-369 (PMC3529689; doi:10.1186/1472-6963-12-369)
Supplement: Additional file 3: Attachment 3 — Sample Kanyini Audit feedback report. [file 1472-6963-12-369-S3.pdf]

Kanyini Audit Study- XYZ Health Service Feedback Report

KANYINI VASCULAR COLLABORATION

**XYZ Health Service**		**National**	
Records audited	200	Records audited	1165
Mean age	42.9	Mean age	41.1
% Male	43.5%	% Male	40.5%
% Female	56.5%	% Female	59.5%

Recorded Past History

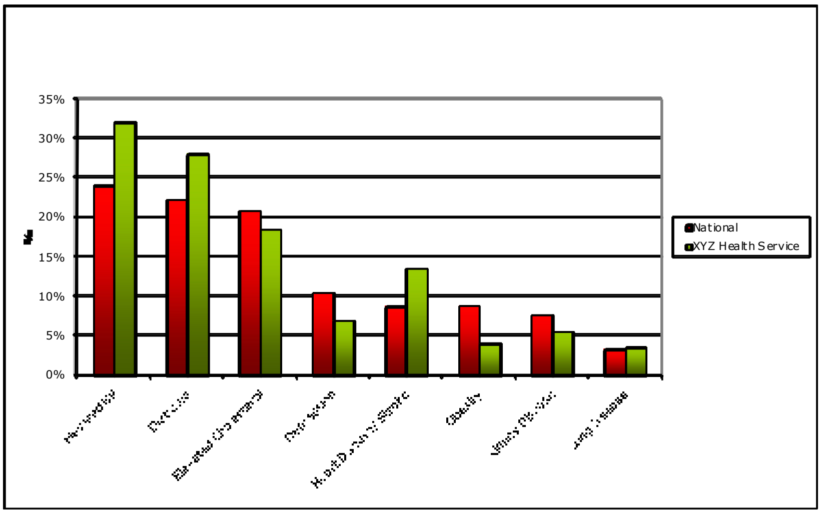

Comment:
These specific recorded conditions were looked for in this audit. The results do not reflect all recorded conditions found in the case record

Risk Factor Recording

Smoking status

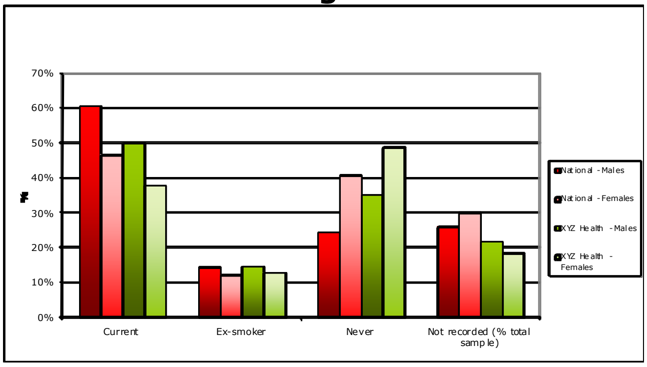

Body Mass Index

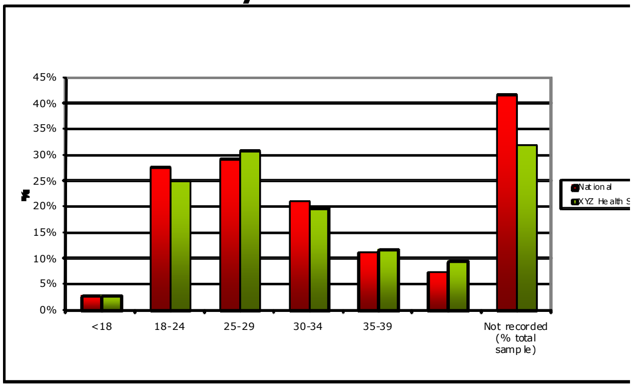

Albuminuria

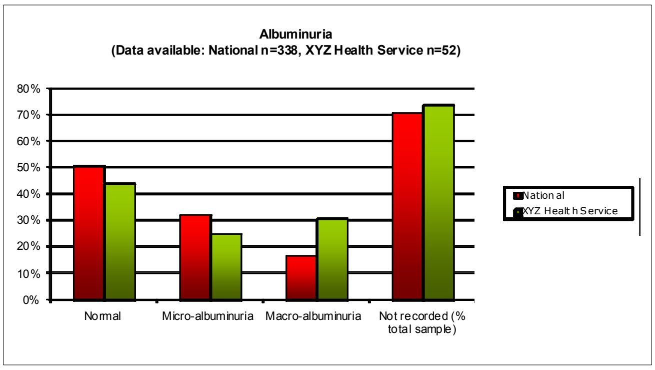

Chronic Kidney Disease

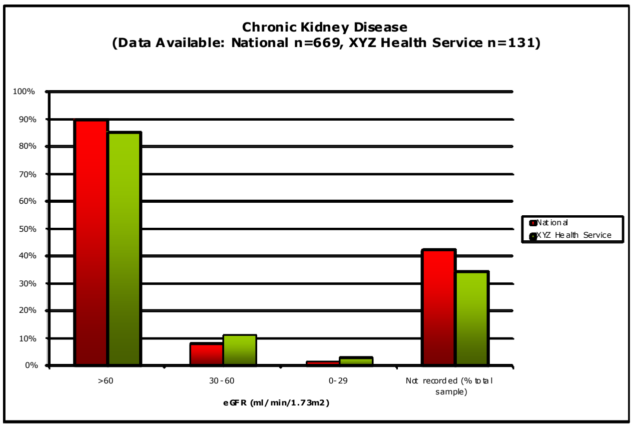

Summary of risk factor recording

	XYZ Health (n=39)	National (n=1165)
**Risk factor recorded**	**%**	**%**
Blood Pressure	98.5%	90.2
Blood glucose	91.5%	78.9
Smoking status	80.0%	71.4
Lipids	67.0%	58.9
eGFR	65.5%	57.4
Body Mass Index (BMI)	59%	55.7
Albumin Creatine Ratio (ACR)	26.0%	29.0
ACR and eGFR for at risk CKD*	32.5%	31.8

* n=151 for XYZ, n=851 for National

Comment:
People 'at risk of Chronic Kidney Disease (CKD)' is based on Kidney Health Australia guidelines and includes any one of the following:

- *Age>50 years*
- *BP >140/90 mmHg*
- *Diabetes*
- *Current smoker*
- *BMI≥30 kg/m^2*

Diabetes Profile

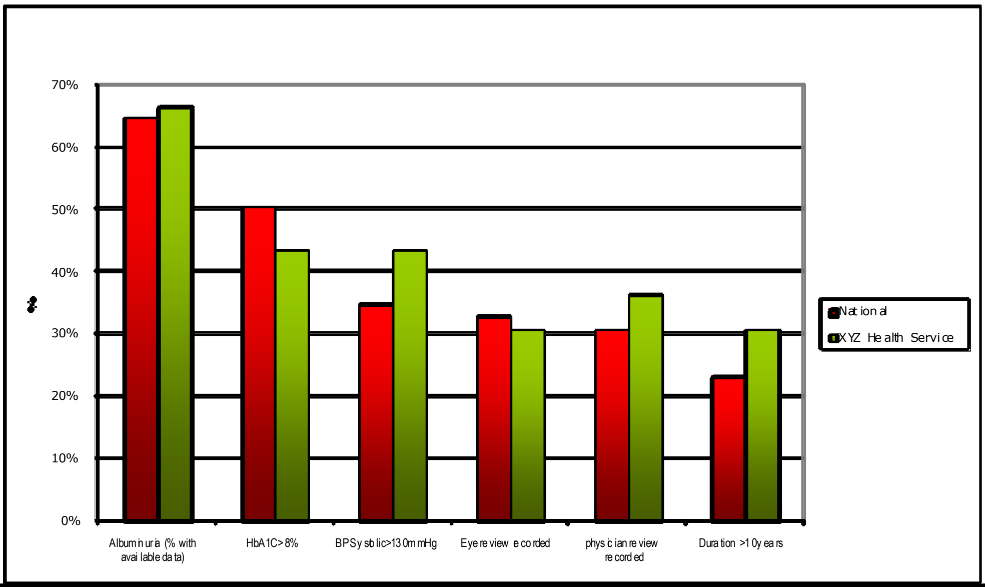

Comment:
Only 36 of 55 people with diabetes had sufficient data to record albuminuria

Cardiovascular risk profile

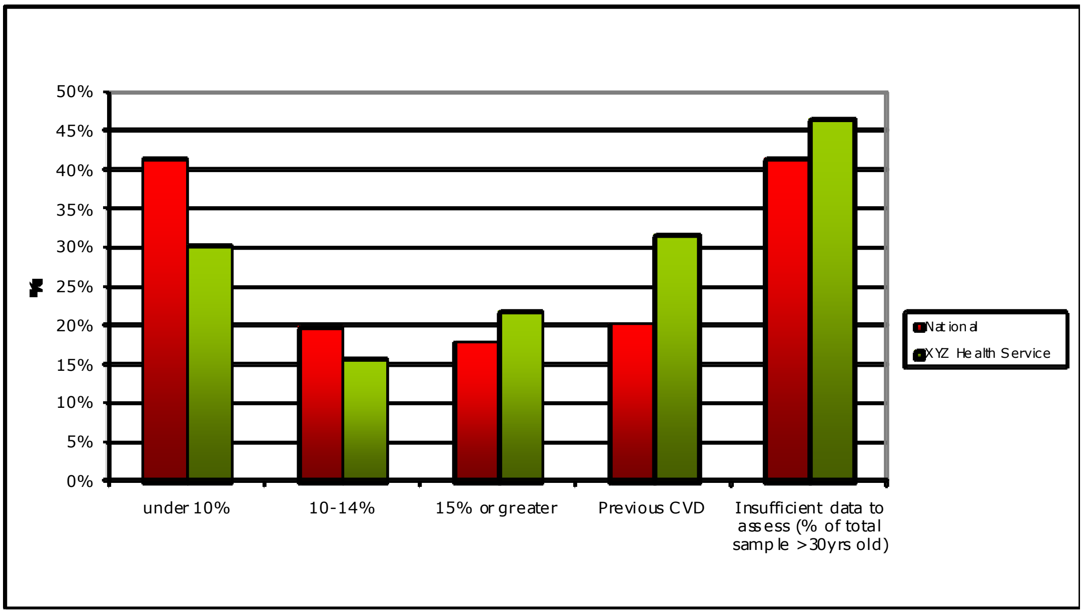

Comment:

Cardiovascular risk is calculated using the Framingham based New Zealand Cardiovascular Guidelines. This includes a 5% addition to the risk calculation for Indigenous populations.

The risk assessment algorithm is not validated for under 30 year olds

**Sufficient data were only available for 82 of 154 people in the sample aged over 30 years old and therefore results should be interpreted with caution*

Care provided

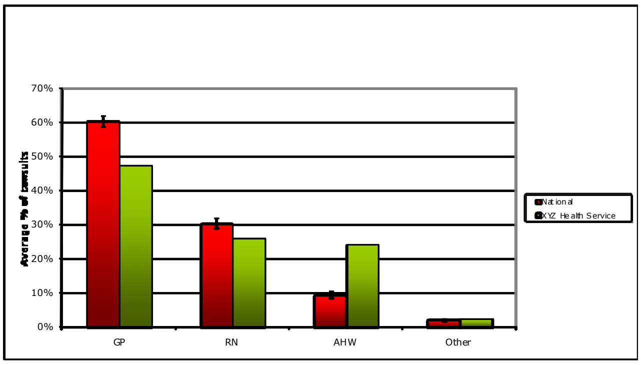

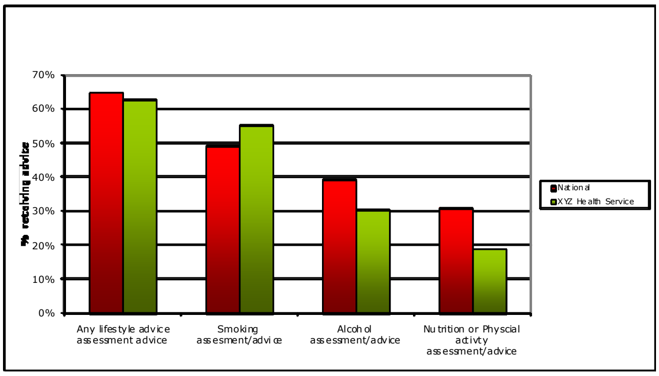

Comment:
Lifestyle assessment was recorded as present if there was any evidence in the clinical record of discussion with the client about smoking, nutrition, physical activity or alcohol in the past 2 years

Enhanced Primary Care Items

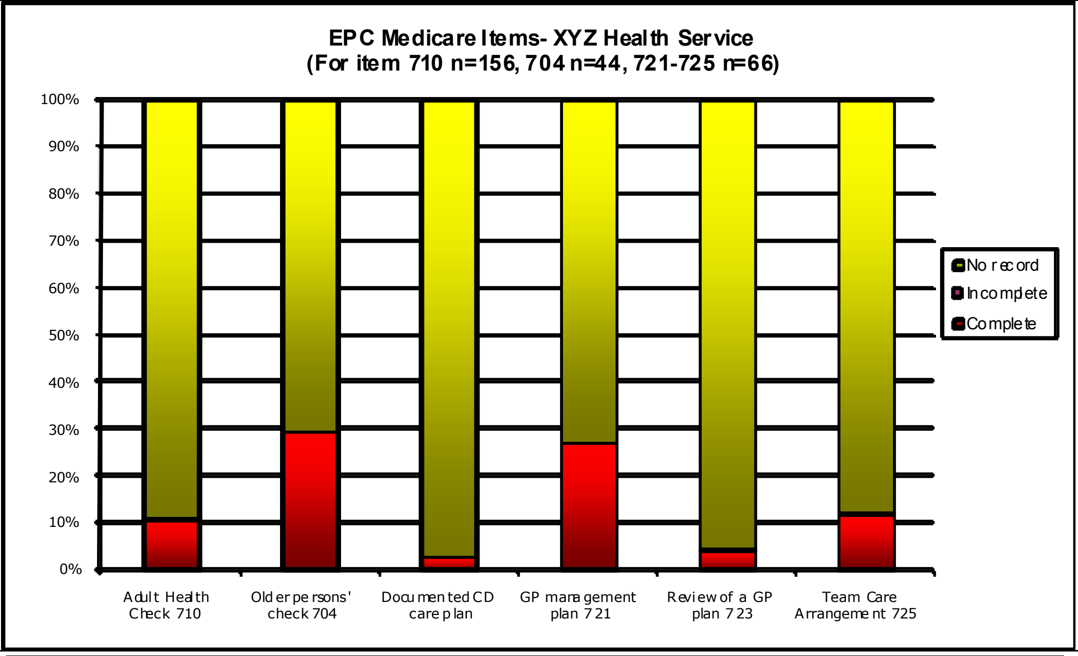

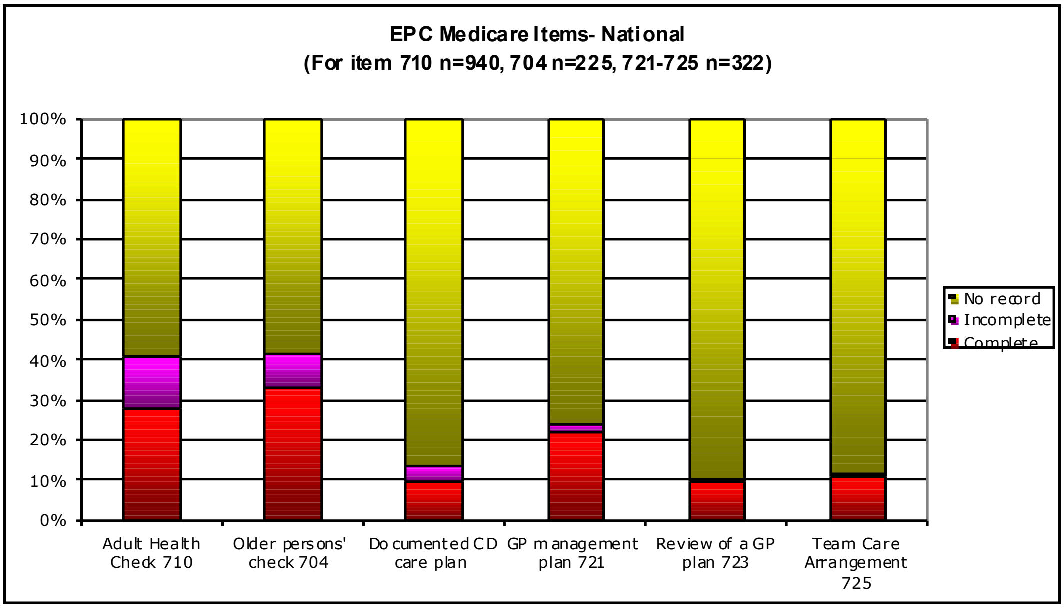

Comment:

EPC items were recorded as complete if there was evidence in the record of a complete assessment and/or evidence of billing for that item. If there was evidence of an unclaimed or partially filled assessment it was recorded as incomplete.

Cardiovascular medicines prescribed

	Total Sample		Clients with Diabetes*		Clients with CVD*	
	XYZ n=200	National n=1165	XYZ n=55	National n=249	XYZ n=27	National n=101
**Medications**	**%**	**%**	**%**	**%**	**%**	**%**
Blood pressure lowering	30%	28	66%	76	82%	81
Cholesterol lowering	27%	22	69%	64	74%	77
Antiplatelet medication	18%	21	44%	62	63%	74
BP + Statin + Antiplatelet	11%	14	29%	42	44%	58

Blood pressure management- prescribing patterns

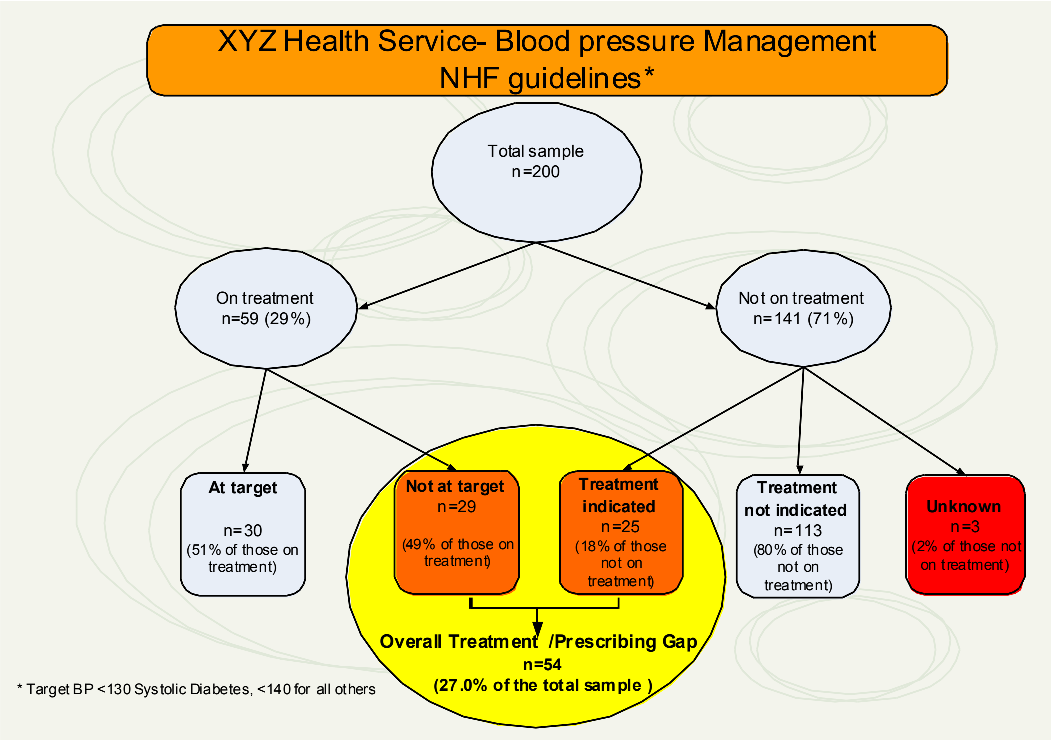

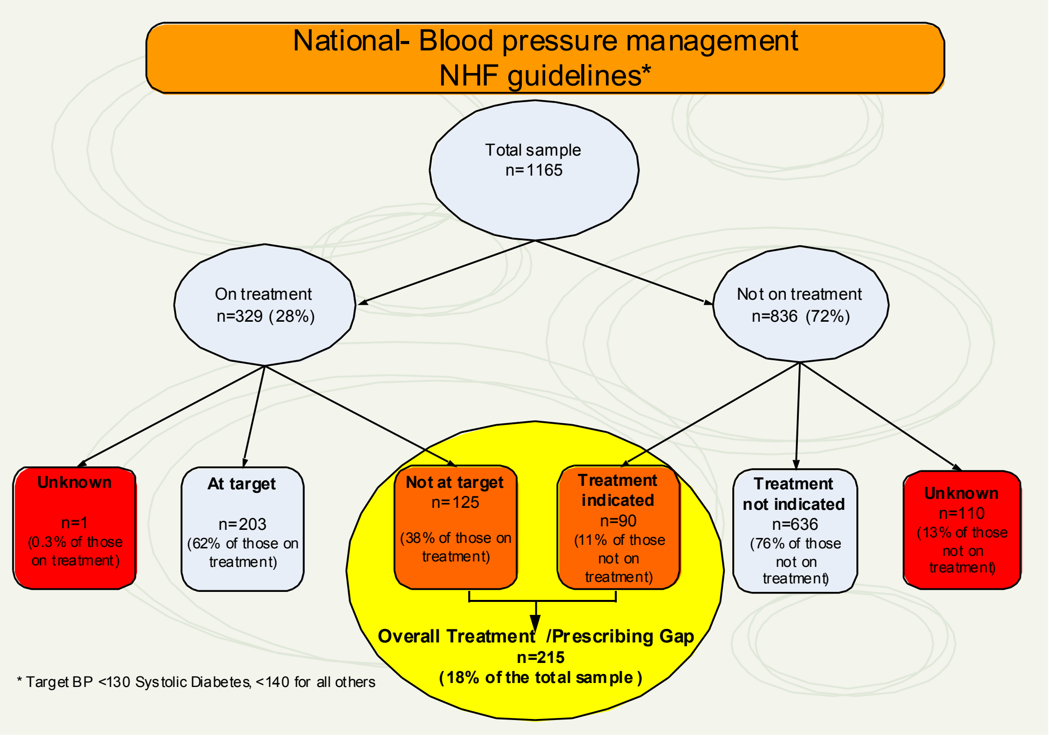

Comment:

Treatment thresholds were based on the average of the two most recent systolic blood pressure recorded values

Treatment indications and target values were measured against the National Heart Foundation guidelines

Lipid management- prescribing patterns

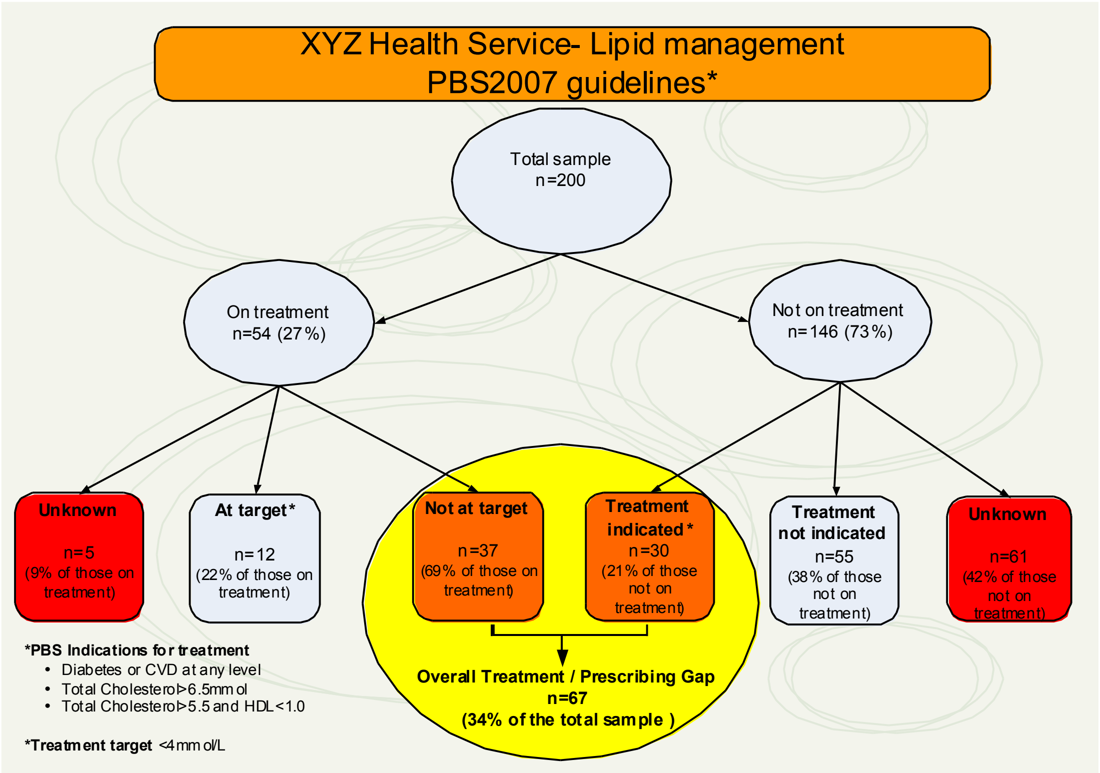

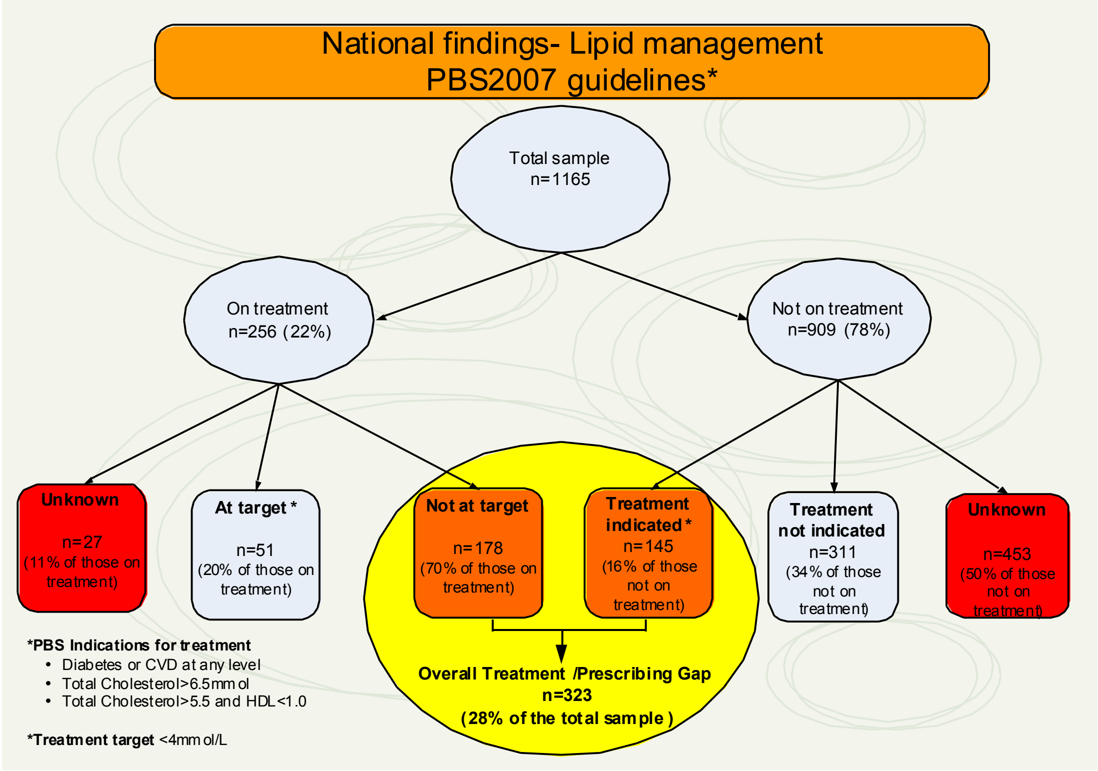

Comment:

Treatment thresholds were based on the most recent cholesterol results.

Treatment indications and target values were measured against the 2007 Pharmaceutical Benefits Scheme indications for Aboriginal and Torres Strait Islander people.
